# Pattern of biopsy proven renal diseases at PNS SHIFA, Karachi: A cross-sectional survey

**DOI:** 10.12861/jrip.2013.43

**Published:** 2013-10-10

**Authors:** Sohail Sabir, Muhammed Mubarak, Irfan Ul-Haq, Aisha Bibi

**Affiliations:** ^1^Departments of Nephrology1and Histopathology2, PNS Shifa and SIUT, Karachi, Pakistan

**Keywords:** Epidemiology, Renal biopsy, Glomerulonephritis, Nephropathy

## Abstract

**Introduction:** Percutaneous renal biopsy (RB) is an invaluable diagnostic procedure in patients with medical renal diseases.Objectives: To determine the pattern of biopsy proven renal disease (BPRD) from a tertiary care naval hospital in Karachi, Pakistan.

** Methods and Materials:** All the renal biopsies in adult patients (≥18 years) performed at our hospital from 2008 to 2012 were retrospectively reviewed. The biopsies were evaluated by light microscopy and immunofluorescence.

** Results:** A total 60 cases were analyzed. The mean age was 33.3±12.9 years (range: 18 to 72 years).The male to female ratio was 3:1. The most common indication of renal biopsy was nephrotic syndrome (43.3%), followed by renal failure (26.6%) and non-nephrotic proteinuria (23.3%). Primary glomerulonephritides (PGN) were predominant overall lesions, found in 46 (76.6%) of the total biopsies. Among PGN, the most common lesion was focal segmental glomerulosclerosis (FSGS), followed by membranous glomerulonephritis (MGN), IgA nephropathy (IgAN) and chronic sclerosing glomerulonephritis (CSGN) and a variety of rare lesions. Secondary glomerulonephritides (SGN) were found in only three (5%) cases. There were two cases of amyloidosis and one of lupus nephritis (LN). Tubulointerstitial disease (TID) and vascular disease were rare.

** Conclusion:** This study provides information about the epidemiology of BPRD in a large tertiary care naval center in Southern Pakistan.

**Implication for health policy/practice/research/medical education:**

Retrospective reviews and analysis of renal biopsies serve a useful function in understanding the epidemiology of medical renal diseases in an area. Although, inherently biased and heavily dependant on biopsy policies, these studies do shed some light on the prevalent renal diseases in a community or region. This study represents such an attempt of determining the most common medical renal diseases in adults from this part of the world.

## Introduction

Percutaneous renal biopsy (RB) is an invaluable diagnostic procedure in patients with medical renal diseases (1). However, the indications of RB and the extent of its pathological evaluation vary from country to country and even from center to center (2,3). Although the analysis of data obtained from RB is not ideal for understanding the epidemiology of renal diseases, it is nevertheless important for identifying the prevalent patterns of renal disease in a certain area (4).

The prevalence of biopsy proven renal diseases (BPRDs) varies according to the geographic region, socioeconomic conditions, race, age, demography and indication of renal biopsies (5-15). Unfortunately, there is no central renal biopsy registry in Pakistan. Population based studies on the prevalence of renal disease in Pakistan are non-existent (4). Very few studies on BPRD have been published from Pakistan and the neighboring countries (2,4,5-10) and some of these have demonstrated an increase in the incidence of focal segmental glomerulosclerosis (FSGS). Moreover, growing evidence from different published studies across the world indicates a changing pattern of glomerular disease over the last few decades (6,11-24). PNS SHIFA is a tertiary care naval hospital situated in Karachi. The hospital receives patients from southern provinces of Sindh and Balochistan. However, our patients represent mixed demographic data since armed forces personnel and their families from all over Pakistan are posted to our catchment area in addition to the natives. At PNS SHIFA, a nephrologist (SS) performs the renal biopsies along with a radiologist under ultrasound guidance in the radiology department. Till date, we have completed four years of collection of RB data at our hospital, prompting us to carry out this study to understand the pattern of BPRD in our institute which will inform nephrologists and pathologists in future management of such cases.

## Objectives

This study is the first to report pattern of diagnosis of renal biopsies from any armed forces hospital in Pakistan.

## Methods and Materials

All the kidney biopsies performed at our Hospital from January 2008 to July 2012 were retrospectively analyzed from request forms, admission records and biopsy reports. The biopsies were carried out at PNS and interpreted at Histopathology Department of Sindh Institute Of Urology and Transplantation (SIUT), Karachi, Pakistan. The following data items were recorded for each patient: age, sex, indication for RB, histopathological diagnosis and relevant laboratory tests such as serum creatinine, 24 hours urinary protein, virology (HBsAg, anti HCV, HIV) and serology (anti-ds DNA antibody, ANA, C3, C4). The RB specimens obtained were processed and prepared according to standard protocols and examined by a pathologist (MM) with special interest and expertise in nephropathology, as described in our previous study (4). The histopathological evaluation included light microscopy (LM) and immunofluorescence (IF). For LM, 3 sections were stained with Hematoxylin and Eosin (H&E), two with periodic acid Schiff (PAS), one with Masson’s trichrome, and one with Jones silver methenamine. Further special stains were used as and when required. IF study was done by using polyclonal antibodies against human IgG, IgM, IgA, C3, and C1q (Dako, Glosstrup, Denmark). The indications for renal biopsy were classified into five clinical syndromes: nephrotic syndrome (NS), non-nephrotic proteinuria (NNP), proteinuria-hematuria, renal failure (RF), and rapidly progressive glomerulonephritis (RPGN). In RF, RB was performed for unexplained RF if kidney sizes were within normal limits. Standard definitions were used for all these biopsy indications (4). Hypertension was considered when blood pressure was higher than 140/90 mmHg. All the biopsies were obtained by percutaneous automated biopsy gun. Written informed consent was obtained prior to obtaining the biopsies in all cases. Standard histological classification was used to diagnose the pathological entities (25).

### Ethical issues

The research followed the tenets of the Declaration of Helsinki; written informed consent was obtained; and the research was approved by ethical committee of PNS Shifa and SIUT centers.

### Statistical analysis

Statistical analysis was carried out using IBM compatible SPSS for windows version 13 (SPSS Inc., Chicago, IL, USA). Simple descriptive statistics such as mean±SD were used for continuous variables such as age and clinical and laboratory features. Numbers (percentages) were used for categorical variables.

## Results

### Patient characteristics

The demographic characteristics of patients are shown in Table 1. A total of 60 patients with RBs were analyzed retrospectively from 2008 to 2012. Only one biopsy was inadequate, rest all yielded adequate tissue for proper diagnosis and classification of underlying diseases. One patient had dual pathology on RB. Among these, 45 (75%) were males and 15 (25%) were females. The male to female ratio was 3:1. The mean age of patients was 33.3±12.9 years (range: 18-72 years).

**Table 1:** Demographic characteristics

Total number of patients	60
Males	45 (75%)
Females	15 (25%)
Male to female ratio	3:1
Mean age (in years)	33.3 ± 12.9
Age range (in years)	18–72

The indications for RBs in our study are shown in Table 2 . As is evident from this table, the most common indication for renal biopsy was NS, followed by RF, and NNP. Other indications were rare.

**Table 2:** Clinical indications for renal biopsies in 60 adults with medical renal diseases

**Clinical indications**	**Number**	**Percentage**
Nephrotic syndrome	26	43.3
Renal failure	16	26.6
Non-nephrotic proteinuria	14	23.3
Non-nephrotic proteinuria, hematuria	2	3.3
Rapidly progressive glomerulonephritis	2	3.3
Total	60	100.00

### Pathologic Findings

The frequencies of different renal diseases in percutaneous native renal biopsies in adult patients at our hospital are shown in Table 3 . It is evident from this table that the glomerular diseases were most prevalent and among these PGD were overwhelmingly predominant. SGN were less frequent as were TID and vascular diseases. Table 4  shows a comparison of some important renal diseases found in our study with a few previously published local, regional and international studies. It is obvious from this table that the pattern of BPRD observed in our study closely resembles that reported previously from Pakistan. There are however some differences when compared with regional and international studies.

**Table 3:** Breakdown of renal biopsy diagnoses in 60 renal biopsies from adult patients.

**Renal Diseases**	**Number**	**Overall percentage**
Primary GN	46	76.6
FSGS	16	26.6
Membranous GN	10	16.6
IgAN	7	11.6
Chronic sclerosing GN	6	10
Minimal change disease	3	5
Postinfectious GN	1	1.6
IgMN	1	1.6
MPGN	1	1.6
C1q nephropathy	1	1.6
Secondary GN	3	5
Lupus nephritis	1	1.6
Amyloidosis	2	1.6
Tubulointerstitial disease	7	11.6
Tubulointerstitial nephritis	4	6.6
Tuberculosis	3	5
Vascular Disease	4	6.6
Acute cortical necrosis	1	1.6
Benign nephrosclerosis	2	3.3
Vasculitides	1	1.6
Total	60	100
FSGS, focal segmental glomerulosclerosis, IgAN, IgA nephropathy, IgMN, IgM nephropathy, MPGN, membranoproliferative GN.

**Table 4:** Comparison of some common diseases in our series with other studies (all figures are in percentages).

**Diseases **	**Our study**	** Pakistan (4) **	** India (7) **	** UAE (8) **	** Saudi Arabia (9) **	** Italy (12) **
Glomerulonephritides (GN)	81.6	83.58	71	-	76	-
Primary GN	76.6	86.57	71	77.1	68	59.95
FSGS	26.6	20.89	17	18.3	21.8	11.8
Membranous GN	16.6	16.97	9.8	20.1	21.8	20.7
Minimal change disease	5	5.52	11.6	18.3	26.4	7.8
IgA nephropathy	11.6	1.49	8.6	6.3	1.9	34.5
Secondary GN	5	14.4	-	16.5	32	25.4
Amyloidosis	1.6	4.69	-	33.3	32	-
Lupus nephritis	1.6	4.80	6.5	40.7	-	-
Diabetic nephropathy	-	20	-	1.10	-	-
Tubulo-interstitial disease	11.6	11.22	2.5	-	-	4
Vascular disease	1.6	3.92	-	-	-	33

## Discussion

Although there are several limitations in the study, it provides important information about the demographics, clinical syndromes and the pattern of kidney diseases diagnosed by RB during a period of four years at a single tertiary care referral institute in Karachi, Pakistan. The main limitations of the study include its retrospective nature, single center based study, relatively small size of the sample, and somewhat biased biopsy indications in favor of more severe kidney diseases. This makes direct comparisons with different published studies and accurate conclusions somewhat difficult. Nevertheless, it provides a significant insight into the pattern of BPRD prevalent in the catchment area of our hospital.

The demographic and clinical characteristics of our cohort are more or less similar to those reported in other regional studies (4-10). We observed a male predominance in all cases of renal diseases as well as the predominant age affected is the young adult group and that is because our patients in majority belong to armed forces. Similar to other studies, our data showed that nephrotic syndrome was the most frequent clinical presentation in all age groups, accounting for 43.3% of all cases (4,6,9). On the other hand, studies from Japan and Europe reported a higher frequency of minor urinary abnormalities as biopsy indications with consequent differences in the histopathological lesions (11-21).

The underlying pathology of NS is highly variable across the world. In our study, the most common pathological lesion underlying NS was membranous GN (MGN), closely followed by FSGS, while MCD, IgAN and MPGN were rare. This is slightly different from that reported from other local and regional studies (4-10). Many studies from Europe however observed similar findings (11-21). The primary glomerulonephritides (PGD) were the most predominant renal diseases in our study as well as in all recent studies, followed by SGN and TIN (4-10,2-19). The vascular diseases were also less frequent in almost all studies. We did not observe any hereditary GN which may be due to lack of electron microscopic (EM) study in the present analysis and also because our armed forces personnel are screened thoroughly for preexisting diseases at the time of induction. Regarding specific glomerulopathies, the prevalence of FSGS in renal biopsy series is highly variable. In addition, during recent years, there is a worldwide increase in the incidence of FSGS (23,24). Overall, it is the most common PGD in the present study. It was also the most common overall pathological lesion in our earlier study (4). MGN is the second most common PGD in this study. However, MGN is more common in NS than FSGS in this series. The older literature cited MGN as the most common PGD in adults, but most recent studies have found a declining prevalence of MGN and a corresponding rise in the prevalence of FSGS in adults. However, MGN is still common in some regions of Asia, Europe and America (16,18-20,24).

IgAN is less prevalent in the present series as well as other studies from this region of the world (4-10). In contrast to this, it is the most common PGD in European countries and some Asian countries (11-21). However, the prevalence is slightly higher than that observed in other studies from Pakistan (4). This may be because of our slightly liberal biopsy policy. Four cases of IgAN with presentation as NNP or NNP-hematuria were biopsied in our study.

We also observed an increase in the incidence of CSGN in our series. Most of these patients presented with RF with normal size kidneys. The relatively high prevalence of this advanced pathology may be due to delayed presentation of patients (4).

The SGN were less frequent in this study. Two cases of amyloidosis and one case of lupus nephritis were observed. Both cases of amyloidosis were secondary in nature. Our results are concordant with the data from other local and regional studies (4,6,8,10). We performed RB only on clinically unsuspected cases of amyloidosis. In cases, where suspicion of amyloidosis was high, it was confirmed by biopsies from other sites such as rectum.

TID is relatively less frequent BPRD in our study as in many previously reported regional studies (4-10). A high number of tuberculosis in the current study is of interest. These cases were first diagnosed on RB.

## Conclusion

The study provides important information on the epidemiology of BPRD from southern part of Pakistan. Our results are slightly different from other local and regional studies, most probably due to varying biopsy indications. There is a need to establish central renal biopsy registry to collect and analyze information on BPRD in this part of the world.

## Authors’ contributions

SS and MM conceptualized the study, obtained and analyzed the data, drafted the paper and gave final approval. IH provided important intellectual input and AB helped in data acquisition and data analysis.

## Conflict of interests

The author declared no competing interests.

## Ethical considerations

Ethical issues (including plagiarism, data fabrication, double publication) have been completely observed by the author.

## Funding/Support

None.
